# Seroprevalence of SARS-CoV-2 in cats from COVID-19 positive households in the Lisbon area

**DOI:** 10.3389/fvets.2025.1542397

**Published:** 2025-06-23

**Authors:** Isa Moutinho, Sara Cardoso, Mafalda Henriques, João Gonçalves, Luís Tavares, Solange Gil, Telmo Nunes, Frederico Aires-da-Silva

**Affiliations:** ^1^Center for Interdisciplinary Research in Animal Health (CIISA), Faculty of Veterinary Medicine, University of Lisbon, Lisbon, Portugal; ^2^Associate Laboratory for Animal and Veterinary Sciences (AL4AnimalS), Lisbon, Portugal; ^3^Research Institute for Medicines (iMed.ULisboa), Faculty of Pharmacy, University of Lisbon, Lisbon, Portugal

**Keywords:** SARS-CoV-2, COVID-19, human-to-cat transmission, antibodies, One Health

## Abstract

**Introduction:**

Since the onset of the COVID-19 pandemic, the transmission of SARS-CoV-2 between humans and cats has been well-documented. However, the dynamics of this cross-species transmission remain insufficiently understood. Seroprevalence studies in cat populations across different contexts and regions are crucial for estimating viral infection rates and tracking viral evolution. Furthermore, identifying risk factors associated with human-to-cat transmission is essential.

**Methods:**

This study, conducted during the COVID-19 pandemic, assessed SARS-CoV-2 transmission and seroprevalence in 76 cats from COVID-19-positive households in the Lisbon area. Antibodies against SARS-CoV-2 variants (alpha, delta and omicron) were detected using enzyme-linked immunosorbent assay (ELISA). Positive samples were further tested for neutralizing antibodies using surrogate virus neutralization test (sVNT) and pseudotyped virus assays. To identify risk factors for human-to-cat SARS-CoV-2 transmission, we analyzed the association between animal characteristics, cat-owner interactions, owner clinical signs and sVNT results.

**Results:**

Of the 76 cats tested, 23 (30.3%) were ELISA-positive for SARS-CoV-2 antibodies. Among these, 16 (69.6%) exhibited potent neutralizing antibodies confirmed via sVNT and pseudotyped virus assays. Compared to our previous study of cats with unknown exposure to SARS-CoV-2 in the Lisbon area, this study found significantly higher rates of seroprevalence (30.3% vs. 14.7%) and neutralizing antibody prevalence (69.6% vs. 20.4%) in cats from COVID-19-positive households. None of the risk factors studied showed a statistically significant association with seropositivity in cats.

**Discussion:**

These findings suggest a greater exposure and infection risk in cats from COVID-19-positive households. The absence of significant associations with the analyzed risk factors highlights the complexity of human-to-cat SARS-CoV-2 transmission. Future studies should further investigate the impact of demographic characteristics, health conditions, lifestyle, owner-cat interactions, and owners’ symptoms during infection to better understand their role in SARS-CoV-2 transmission from humans to cats and to inform strategies for controlling future outbreaks.

## 1 Introduction

Emerging infectious diseases represent a significant and growing threat to global public health, with their incidence steadily increasing in recent years. Approximately 60–75% of emerging infectious diseases have a zoonotic origin, highlighting the urgent need to understand how human activities disrupt ecosystems and increase contact with animal reservoirs of these agents (1, 2). Coronaviruses (CoVs) are prime examples of zoonotic pathogens with pandemic potential. While often circulating harmlessly in animal populations, CoVs possess high adaptability to mutate and evolve, facilitating their ability to transition to new hosts such as humans (3).

The COVID-19 pandemic, caused by severe acute respiratory syndrome coronavirus 2 (SARS-CoV-2), has raised awareness of the risks posed by CoVs and the importance of zoonotic disease surveillance. Furthermore, the COVID-19 pandemic has also highlighted an intricate interconnection between human and animal health. As of December 3, 2024, more than 15 species are noted by the Food and Agriculture Organization of the United Nations (FAO) to be susceptible to natural infection by SARS-CoV-2. Among them, felids (cats and big cats), dogs, minks, ferrets, white-tailed deer and others (4–15). In companion animals, such as cats and dogs, data indicate that prevalence varies greatly based on region and is higher in animals originating from COVID-19 positive households compared with animals possessing unknown exposure status to the pathogen (16, 17). These findings highlight the importance of understanding SARS-CoV-2 transmission dynamics across animal and human populations.

As of December 1, 2021, 14-day cumulative incidence of COVID-19 cases in Lisbon and Tagus Valley had reached 353 cases per 100.000 inhabitants and the trend was increasing (+25% compared to the previous week), according to the Portuguese Directorate-General of Health (DGS) and the National Institute of Health Doutor Ricardo Jorge (INSA). During that month Portugal experienced a recorded in the number of new cases, with Lisbon and the Tagus Valley consistently being one of the most affected regions. In January 2022, Portugal registered over 65.000 new cases in a single day, with a vast majority concentrated in the Lisbon metropolitan area. With these data, urban areas such as Lisbon, with high population density, intense urban mobility and close human-pet cohabitation, providing an ideal context for the study of zoonotic spillover events. This environment offers a valuable opportunity to explore viral spillovers in a real-world scenario.

Cats have become a particular concern due to their close contact with humans and their high susceptibility to SARS-CoV-2 infection, attributable to the virus’s strong affinity for the feline ACE2 receptor, a key component in the viral entry process (18–31). According to published studies, cats can contract SARS-CoV-2 from humans (human-to-cat transmission), typically resulting in asymptomatic or mild infections (32, 33). However, the existence of comorbidities such as intestinal B-cell lymphoma may exacerbate the severity of clinical signs in affected cats (34). Regarding the transmission of the virus from cats to humans, only one suspected case has been described. Thus, at the present, there is no conclusive evidence to support cat-to-human transmission (35). Research has also focused on cat-to-cat transmission, where infected cats, under experimental conditions, have been able to transmit the virus to sentinel cats, showing both viral shedding and seroconversion, which indicates effective transmission (32, 36, 37).

Considering these findings, an emerging concern with SARS-CoV-2 in cats is the potential for the virus to mutate in these animals. Viral adaptation in animals could result in genetic changes that impact transmissibility or virulence, posing additional risks for both human and animal health. Furthermore, the role of cats as potential intermediaries in viral transmission remains uncertain, warranting further investigation.

Although substantial progress has been made in understanding SARS-CoV-2 infection in cats, some questions remain unanswered. Limited information is currently available regarding the risk factors contributing to the transmission of SARS-CoV-2 from humans to cats. Consequently, additional research is necessary to better understand the role of variables such as cat behavior and interactions with humans involved in transmission. In addition, extensive seroprevalence studies are required in various regions and in different cat populations, pets, shelters, strays, and farm/rural cats to assess the prevalence of exposure to SARS-CoV-2 and identify contexts with high risk of transmission.

In a previous study conducted in the Lisbon region, our group investigated SARS-CoV-2 seroprevalence and antibody responses in cats with unknown exposure to the virus (38). The present study, also conducted in the Lisbon metropolitan area, focuses on cats from COVID-19-positive households to assess the seroprevalence of SARS-CoV-2 in these animals and compare the results with our previous data on cats with unknown exposures. Additionally, we aimed to analyze factors such as cats’ age, sex, breed, medical history, lifestyle, cat-owner interactions and owners’ symptoms to enhance our understanding of human-to-cat SARS-CoV-2 transmission.

## 2 Materials and methods

### 2.1 Study population and sample processing

Between November 2021 and March 2022, 76 cats from the Lisbon metropolitan area, living in 50 households with confirmed COVID-19 cases, were enrolled in this study. Owners were informed about the study’s objectives and provided informed consent. They also completed an online questionnaire detailing the cat’s age, sex, breed, medical history (Supplementary Table S1) as well as additional information on lifestyle, cat-owner interactions and owners’ symptoms (Supplementary Tables S2–S4). Sample collection was performed either at the Veterinary Teaching Hospital of the Faculty of Veterinary Medicine at the University of Lisbon (HEV-FMV-ULisboa) or during veterinary house calls. Blood samples were collected, processed, and stored as described by Moutinho et al. (38). The sample size was calculated using the Epitools software, based on an expected prevalence of 20% derived from our previous study (38) and a precision of 10%. This method, as described by Thrusfield (39), was used to ensure sufficient power for subsequent analyses. The study was conducted in accordance with the local legislation and institutional requirements and was approved by Animal Care and Ethical Committee of the Faculty of Veterinary Medicine at the University of Lisbon (Ref No. 00142023/00202025).

### 2.2 Detection of anti-RBD-SARS-CoV-2 antibodies by ELISA

A total of 76 samples were tested for immunoglobulin G (IgG) against SARS-CoV-2 using enzyme-linked immunosorbent assay (ELISA) as described by Moutinho et al. and Castro et al. (38, 40). First, 96-well plates were coated with SARS-CoV-2 receptor-binding domain (RBD) (2 μg/mL) alpha (B.1.1.7), delta (B.1.617.2), and omicron (B.1.1.529) (Abbexa, United Kingdom) and incubated for 1 h at 37°C. After washing, plates were blocked with PBS/2%BSA for 1 h at 37°C. Cat serum samples were added to the wells and incubated for 1 h at 25°C. Plates were washed and anti-cat-IgG-HRP (Abcam, United Kingdom) was added at a 1:10,000 dilution and incubated for an additional hour at 25°C. Finally, ABTS substrate was added and the optical density (OD) was measured at 415 nm using an iMark™ microplate reader. For each serum sample, the OD values were measured for the different variants of SARS-CoV-2 RBD and BSA (control). A cut-off value for positivity was established at 0.5, which was calculated as twice the mean OD value of the BSA control. Serum samples with OD values for at least one of the RBDs equal to or above 0.5 were classified as positive.

### 2.3 Detection of neutralizing antibodies against SARS-CoV-2

ELISA-positive samples were tested for neutralizing antibodies against SARS-CoV-2 using a commercial Surrogate Virus Neutralization test (sVNT) (GenScript, The Netherlands). This assay was based on protein–protein interaction between the SARS-CoV-2 receptor-binding domain (RBD) and the angiotensin-converting enzyme 2 (ACE2), the host cell receptor used for viral entry. Briefly, samples were incubated with HRP-labeled SARS-CoV-2 RBD to allow the binding of RBD-specific antibodies. Next, the mixture was added to a plate coated with the ACE2. The assay was conducted with Wuhan-Hu-1, Alpha (B.1.1.7), Delta (B.1.617.2), and Omicron (B.1.1.529) variants. As recommended by the manufacturer, only samples with at least 30% neutralization for any one of the RBDs were considered positive. As the sVNT assay does not involve live SARS-CoV-2 and is based on a protein–protein interaction (RBD: ACE2), all experiments were conducted under biosafety level 2 (BSL-2) conditions, following institutional biosafety guidelines.

### 2.4 SARS-CoV-2 pseudotyped-based neutralization assays

The SARS-CoV-2 pseudotyped-based neutralization assays were performed as previously described by Moutinho et al. (38). Briefly, HEK293T cells (CRL-1573™, ATCC®) were seeded at 8.5 × 10^5^ cells/well in a six-well cell culture plate in Dulbecco’s modified Eagle’s medium (DMEM) supplemented with 10% fetal bovine serum (FBS) and 1% penicillin–streptomycin and incubated at 37°C with 5% CO2. Cells were transfected using Lipofectamine 2000 (Thermo Fisher Scientific, United States) with a lentiviral transfer plasmid expressing luciferase (117735, Addgene, United States), packaging plasmids (12251 and 12253, Addgene, United States), and plasmids encoding the spike protein of SARS-CoV-2 Wuhan-Hu-1 D614G (plv-cov2-sd19g, InvivoGen, United States). After 48 h, the culture medium was collected and the pseudovirus titers were determined using the HIV p24 ELISA kit (Abcam, United Kingdom). HEK293T-ACE2 cells (hkb-hace2, InvivoGen, United States) were seeded in 96-well cell culture plates and maintained in DMEM medium supplemented with 10% FBS, 1% penicillin–streptomycin, and 0.5 μg/mL puromycin at 37°C with 5% CO_2_. sVNT-positive serum samples and negative control serum (sample with no binding activity against SARS-CoV-2 receptor-binding domain (RBD) or sVNT neutralizing properties) were preincubated with SARS-CoV-2 pseudotyped viruses for 1 h. Subsequently, serum-pseudovirus mixtures with a multiplicity of infection (MOI) of 10 were added to HEK293T-ACE2 cells. Cells were lysed and luciferase activity was measured using the Pierce™ Firefly Luciferase Glow Assay Kit (Thermo Fisher Scientific, United States). The neutralization of the sera was analyzed using GraphPad Prism version 8.0.1 (GraphPad Software, United States). Due to limited serum volume, samples were only tested against SARS-CoV-2 pseudoviruses carrying the Wuhan-Hu-1 D614G spike protein. The SARS-CoV-2 pseudotyped-based neutralization assays do not involve replication-competent viruses. Instead, it uses pseudoviruses carrying the SARS-CoV-2 spike protein to measure neutralizing antibody activity under safer conditions. These experiments were conducted under biosafety level 2 (BSL-2) conditions, in accordance with institutional and international biosafety guidelines.

### 2.5 Assessment of potential risk factors involved in human-animal transmission

A comprehensive database was created using Microsoft Excel® to organize relevant data related to the cats and their owners. For each cat was included sample information (sample number and collection date), demographic data such as sex, age (classified as junior <2 years, adult 2–12 years, geriatric >12 years), breed [PB (purebred) e MX (mixed breed)], origin (shelter, private, or street), health condition [information about retroviral infections, feline infectious peritonitis (FIP), vaccination status for core vaccines and feline leukemia virus (FeLV)], lifestyle (indoor or outdoor), outdoor access and coexistence with other animals. Additionally, information about humans cohabitants was recorded including infection (number of household members infected with SARS-CoV-2), owner-cat interactions (petting, providing lap time, giving licks, playing, sharing food, co-sleeping, changing litter, or no interaction), and owners’ symptoms (fever, cough, runny nose, loss of smell, loss of taste, chills, vomiting, diarrhea, shortness of breath, fatigue, muscle pain, sore throat, headache, chest, abdominal or joint pain, dermatological changes, or asymptomatic). To investigate the association between these factors and sVNT results, we conducted bivariate analyses using the generalized linear model (Glmer) in R software (version 4.4.1). The household factor was considered as a covariate in the analysis. *p*-values were calculated to assess the significance of these associations, and the results were interpreted within a 95% confidence interval (*p* < 0.05).

## 3 Results

### 3.1 Detection of anti-RBD-SARS-CoV-2 antibodies by ELISA

**Figure 1:**
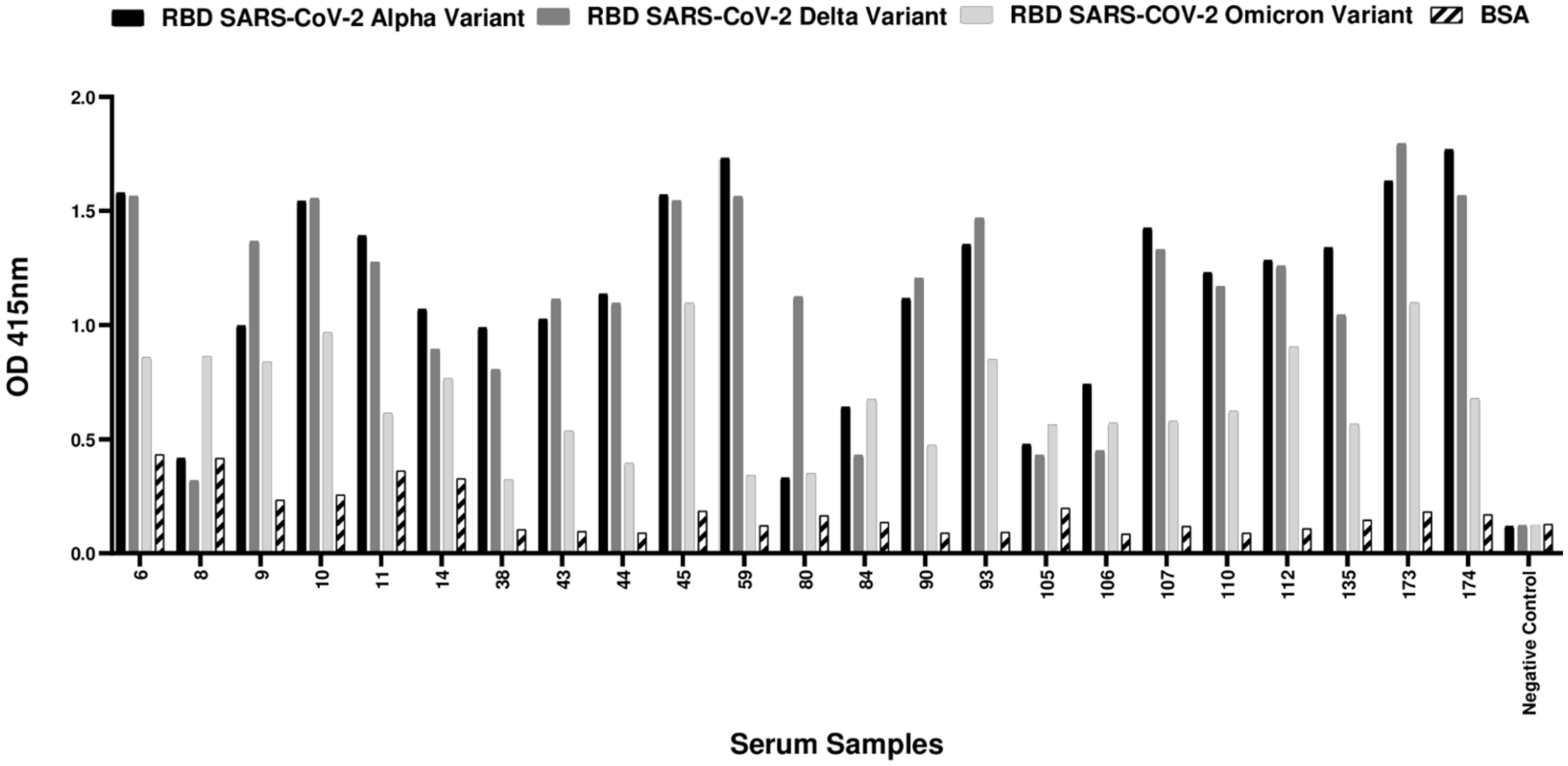
ELISA-positive cat samples against SARS-CoV-2 receptor-binding domain (RBD) proteins in cats from COVID-19 positive households. A total of 76 cat serum samples were collected from households with confirmed COVID-19 cases and tested for antibodies against alpha, delta and omicron SARS-CoV-2 RBD proteins. Among the 76 cat samples tested, 23 (30.3%) had a positive result in ELISA. A cut-off value for positivity was established at 0.5, which was calculated as twice the mean OD value of the BSA control. Serum samples with OD values for at least one of the RBDs equal to or above 0.5 were classified as positive. The negative control consisted of a serum sample collected during the pandemic period, devoid of detectable binding activity against any of the SARS-CoV-2 RBD variants or sVNT virus neutralization properties.

The sera were tested for SARS-CoV-2 receptor-binding domain (RBD)-specific IgG antibodies against alpha, delta and omicron variants by ELISA. Of the 76 samples, 23 (30.3%) tested positive for IgG antibodies against at least one RBD variant (Figure 1).

### 3.2 Detection of neutralizing antibodies against SARS-CoV-2

**Figure 2:**
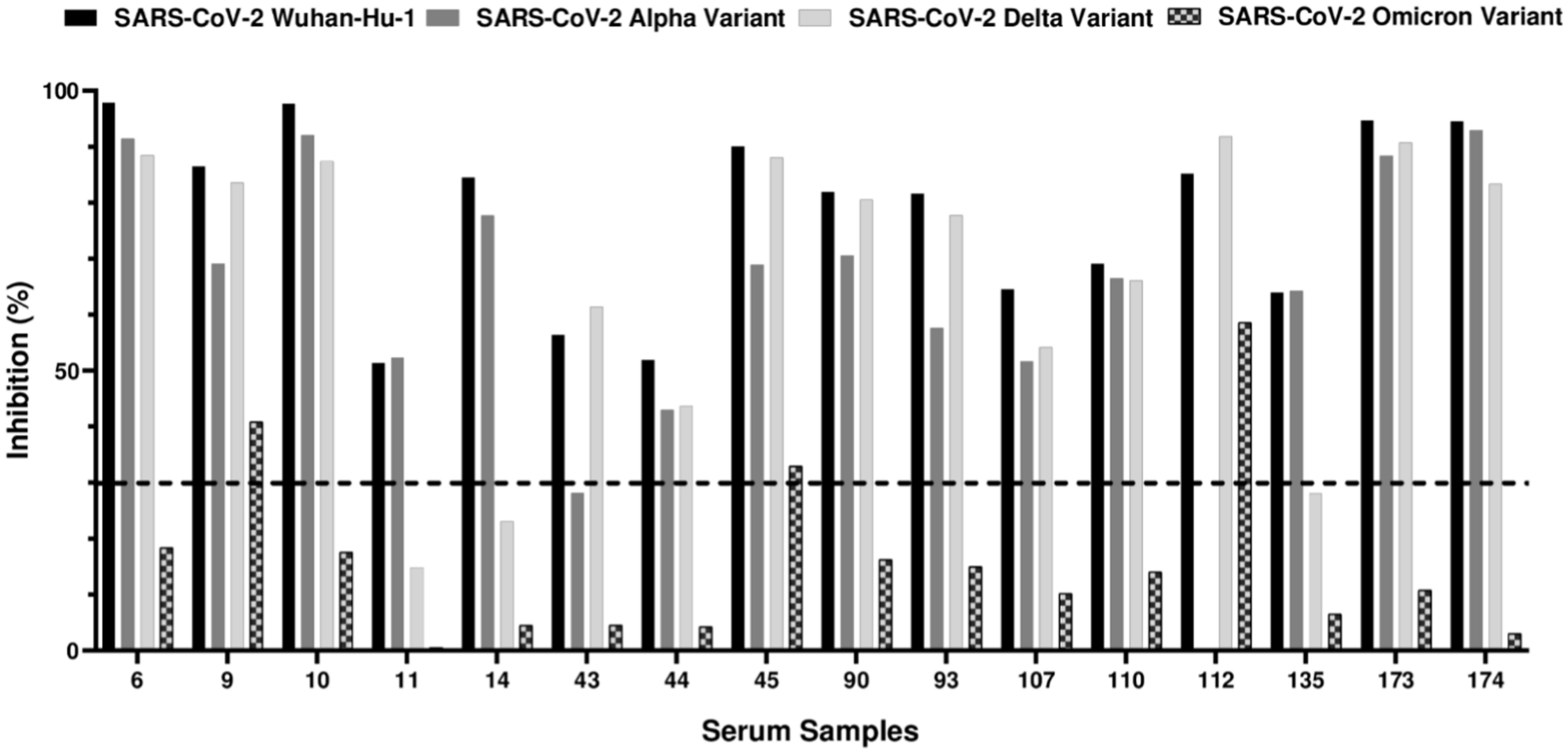
sVNT results against SARS-CoV-2 receptor-binding domain (RBD) from Wuhan-Hu-1 isolate, alpha, delta, and omicron variants. The ELISA-positive samples were evaluated for their ability to neutralize SARS-CoV-2 Wuhan-Hu-1 isolate, alpha, delta and omicron variants using sVNT assay. Out of 23 ELISA-positive samples tested, 16 (69.6%) exhibited neutralizing antibodies against at least one of the variants. The dashed line indicates the 30% neutralization threshold recommended by the manufacturer. Sample 43, while not reaching the 30% cut-off, demonstrated 28.19% RBD-ACE2 inhibition against the SARS-CoV-2 alpha variant and was considered positive. The same procedure was applied to sample 135, which exhibited 28.06% of RBD-ACE2 inhibition against SARS-CoV-2 delta variant and was also considered positive. Sample 112 could not be tested for alpha variant due to insufficient serum.

To evaluate the neutralizing activity of cat IgG antibodies against SARS-CoV-2 variants and verify that the detected antibodies specifically targeted the virus, the 23 ELISA-positive serum samples were subjected to a Surrogate Virus Neutralization Test (sVNT). This assay assessed the ability of serum antibodies to neutralize the SARS-CoV-2 Wuhan-Hu-1 isolate, as well as the alpha, delta, and omicron variants. The test aimed to identify neutralizing antibodies that recognize the SARS-CoV-2 receptor-binding domain (RBD) and inhibit its binding to solid-phase ACE2. Notably, 69.6% (16/23) of the ELISA-positive samples were sVNT-positive, indicating the presence of antibodies capable of blocking the RBD-ACE2 interaction. As shown in Figure 2, 16 out of 23 ELISA-positive samples neutralized both the ancestral SARS-CoV-2 Wuhan-Hu-1 isolate and alpha variant. For the delta variant, all samples, except two (11 and 14), showed detectable neutralizing antibodies. The results for the omicron variant differed significantly; only three samples (9, 45, and 112) exhibited neutralizing activity. These findings suggest a broad neutralization of the ancestral, alpha and delta variants in most samples. The 16 sVNT-positive samples were then subjected to neutralization assays using SARS-CoV-2 pseudotyped viral particles to further validate their neutralizing capacity.

### 3.3 SARS-CoV-2 pseudotyped-based neutralization assays

**Figure 3:**
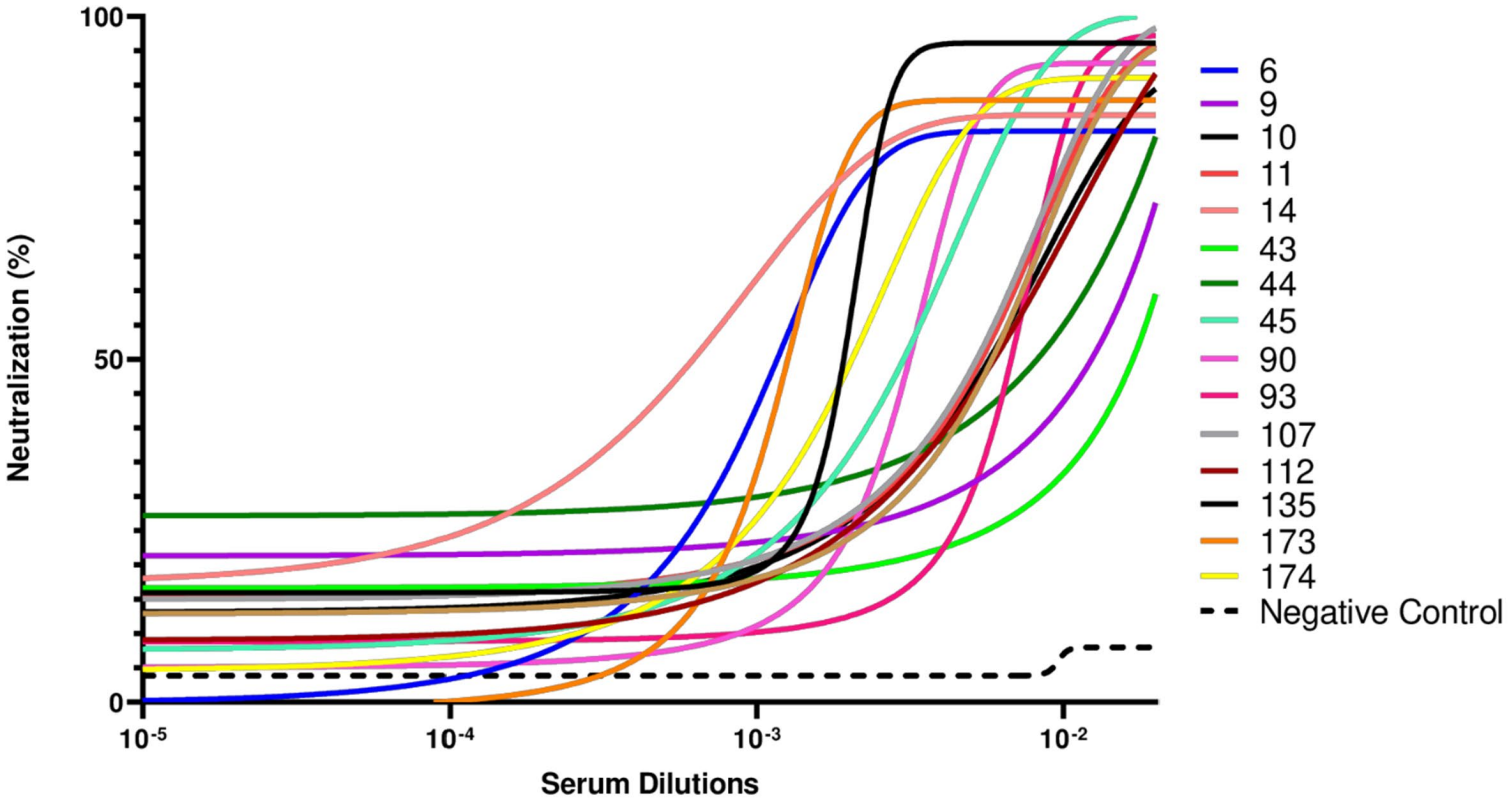
Pseudotyped virus neutralization assay conducted against the SARS-CoV-2 Wuhan-Hu1 D614G variant. This assay was performed to evaluate the neutralizing capacity of the 16 sVNT-positive serum samples to inhibit infection caused by the SARS-CoV-2 Wuhan-Hu-1 D614G variant. The negative control serum, a sample obtained during the pandemic period and with no binding activity against SARS-CoV-2 receptor-binding domain (RBD) from alpha, delta, or omicron proteins and sVNT virus neutralization properties, demonstrated no neutralization. Sample 110 could not be tested due to insufficient serum volume.

To further validate the neutralizing activity of the antibodies in the 16 sVNT-positive samples, we employed a SARS-CoV-2 pseudovirus-based neutralization assay. This approach uses pseudotyped viral particles to more closely mimic viral infection, providing a robust platform for evaluating antibody efficacy in inhibiting the infection process. Due to the limited volume of available serum samples, only SARS-CoV-2 pseudoviruses carrying the Wuhan-Hu-1 D614G spike protein were used in the assay. By transfecting HEK293T cells, we generated pseudotyped lentiviral particles displaying the spike protein of SARS-CoV-2 Wuhan-Hu-1 D614G. After harvesting and titrating, SARS-CoV-2 pseudotyped particles were incubated with serial dilutions of the 16 sVNT-positive samples. After 1 h, the mixture of pseudotyped particles and sera was added to the HEK293T-ACE2 cells. As shown in Figure 3, samples that previously exhibited RBD-ACE2 blocking properties in the sVNT assay effectively neutralized the infection process mediated by the SARS-CoV-2 Wuhan-Hu-1 D614G pseudotyped viral particles. In contrast, the negative control serum, which lacked binding activity against SARS-CoV-2 RBDs or sVNT neutralization properties, showed no evidence of infection neutralization.

Of the 23 samples initially identified as positive by ELISA, 16 demonstrated the ability to inhibit infection in both the sVNT and pseudotyped virus assays, strongly supporting the animals’ exposure to SARS-CoV-2 and the development of virus-specific antibodies. Subsequently, statistical analyses were performed to evaluate the potential association between the sVNT results and specific risk factors that may be related to SARS-CoV-2 human-to-cat transmission.

### 3.4 Assessment of potential risk factors involved in human-cat transmission

The 16 positive samples were analyzed to evaluate the potential association between sVNT results and the animals’ demographic characteristics, owner-animal interactions, and symptoms exhibited by the owners. This analysis aimed to identify risk factors associated with SARS-CoV-2 human-to-cat transmission. A logistic regression model was used to evaluate the association between the dependent variable (sVNT results) and the independent variables (risk factors). Bivariate analysis showed no statistically significant association between the various risk factors studied and sVNT results. However, as shown in Table 1, none of the risk factors studied showed a statistically significant association with seropositivity in cats.

**Table 1:** Association between cat demographic characteristics, owner-cat interactions and owner’s’ symptoms and sVNT results.

Risk factors	Seropositive	Seronegative	Bivariate analysis	
			*p*-value	OR (CI 95%)
Sex				
Male	5/76	31/76	0.1656	0.097 [0.004–2.619]
Female	11/76	29/76		
Age				
Junior	5/76	21/76	0.3752	0.056 [9.674 × 10^−5^ − 32.602]
Adult	10/76	34/76	0.3530	0.138 [2.126 × 10^−3^ − 8.995]
Geriatric	1/76	5/76		
Breed				
Purebred	1/76	4/76	0.4134	0.104 [0.0005–23.453]
Mixed breed	15/76	56/76		
Origin				
Private	5/76	16/76	0.4590	3.673 [0.117–114.993]
Other	11/76	44/76		
Retrovirus				
Positive	-	1/76	0.7389	1.913 [0.042–86.750]
Negative	13/76	42/76	1.000	2.458 × 10^−10^ [0.000 − Inf]
Unknown	3/76	17/76		
FIP				
Positive	-	-	0.7369	1.797 [0.059–54.847]
Negative	13/76	43/76		
Unknown	3/76	17/76		
Core vaccination				
Yes	10/76	33/76	0.741	0.578 [0.022–14.953]
No	6/76	27/76		
FeLV vaccination				
Yes	9/76	18/76	0.4394	2.935 [0.192–44.988]
No	7/76	42/76		
Lifestyle				
Indoor	16/72	58/76	0.946	2.800 × 10^10^ [9.622 × 10^−294^ − Inf]
Outdoor	-	2/76		
Outdoor access				
Yes	1/76	13/76	0.3162	0.073 [0.0004–12.154]
No	15/76	47/16		
Sharing space with other animals				
Yes	14/76	43/76	0.2736	11.961 [1.407 × 10^−1^ – 1016.415]
No	2/76	17/76		
Infected people in the household				
1	3/76	27/76	0.2562	8.467 [2.120 × 10^−1^ − 338.199]
>1	13/76	33/76		
Owner-cat interactions				
Petting				
Yes	15/76	53/76	0.631	4.122 [1.279 × 10^−2^ − 1328.815]
No	1/76	7/76		
Providing lap time				
Yes	15/76	50/76	0.398	9.921 [4.856 × 10^−2^ − 2026.646]
No	1/76	10/76		
Giving licks				
Yes	13/76	32/76	0.2031	12.509 [2.555 × 10^−1^ − 612.369]
No	3/76	28/76		
Playing				
Yes	16/76	51/76	0.983	7.722 × 10^10^ [0 − Inf]
No	-	9/76		
Sharing food				
Yes	6/76	8/76	0.1775	32.436 [2.064 × 10^−1^ − 5097.068]
No	10/76	52/76		
Co-sleeping				
Yes	16/76	44/76	0.964	9.442 × 10^9^ [0 − Inf]
No	-	16/76		
Change Litter				
Yes	15/76	44/76	0.1794	21.626 [2.432 × 10^−1^ − 1922.682]
No	1/76	16/76		
No interaction				
Yes	-	1/76	0.9877	1.929 × 10^−10^ [0 – Inf]
No	16/76	59/76		
Owners’ symptoms				
Fever				
Yes	15/76	31/76	0.1060	1.528 × 10^2^ [3.433 × 10^−1^ − 68049.173]
No	1/76	29/76		
Cough				
Yes	15/76	44/76	0.264	15.706 [1.249 × 10^−1^ 1974.153]
No	1/76	16/76		
Runny nose				
Yes	11/76	31/76	0.457	3.706 [1.175 × 10^−1^ − 116.903]
No	5/76	29/76		
Loss of smell				
Yes	11/76	18/76	0.1117	42.036 [4.200 × 10^−1^ − 4207.278]
No	5/76	42/76		
Loss of taste				
Yes	11/76	20/76	0.1371	36.236 [3.188 × 10^−1^ − 4119.214]
No	5/76	40/76		
Chills				
Yes	8/76	23/76	0.1020	3.220 × 10^6^ [5.102 × 10^−2^ − 2.032 × 10^14^]
No	8/76	37/76		
Vomiting				
Yes	1/16	8/76	0.6473	0.265 [0.0008–78.449]
No	15/76	52/76		
Diarrhea				
Yes	3/76	17/76	0.9044	0.786 [0.015–40.060]
No	13/76	43/76		
Shortness of breath				
Yes	5/76	9/76	0.4537	4.699 [0.082–269.016]
No	11/76	51/76		
Fatigue				
Yes	15/76	33/76	0.1090	69.053 [3.890 × 10^−1^ − 12258.186]
No	1/76	27/76		
Muscle pain				
Yes	11/76	31/76	0.3649	5.806 [1.293 × 10^−1^ − 260.693]
No	5/76	29/76		
Sore throat				
Yes	10/76	33/76	0.6303	2.321 [0.075–71.581]
No	6/76	27/76		
Headache				
Yes	12/76	36/76	0.2658	12.117 [1.497 × 10^−1^ − 980.756]
No	4/76	24/76		
Chest pain				
Yes	1/76	9/76	0.2747	0.060 [0.0003–9.346]
No	14/76	51/76		
No response	1/76	-		
Abdominal pain				
Yes	5/76	9/76	0.3373	9.979 [0.091–1096.384]
No	11/76	51/76		
Join pain				
Yes	6/76	15/76	0.4132	5.414 [0.095–309.519]
No	10/76	45/76		
Dermatological changes				
Yes	-	3/76	1.000	5.329 × 10^−11^ [0.00 − Inf]
No	16/76	57/76		
Asymptomatic				
Yes	-	6/76	1.000	9.798 × 10^−11^ [0.00 − Inf]
No	16/76	54/76		

This table presents the association between the demographic characteristics, owner-cat interactions and owners’ symptoms from the 76 cats included in the study and their results in sVNT test. The table also includes *p*-values and odds ratios (OR) with 95% Confidence Intervals (CI) to demonstrate the statistical significance of the associations between the analyzed factors and sVNT results.

## 4 Discussion

The COVID-19 pandemic, caused by SARS-CoV-2, has raised concerns about viral transmission between humans and pets, particularly cats, due to their high susceptibility to infection and close contact with humans. Seroprevalence studies have been conducted globally to estimate the extent of SARS-CoV-2 infection within this feline subpopulation. Despite numerous efforts, the dynamics of viral transmission between humans and cats remain poorly understood. Specifically, it is unclear whether the transmission of the virus is related to the animal’s lifestyle, type of interaction with the owner or severity of the disease exhibited by the owner. In this context, it is essential to continue characterizing SARS-CoV-2 transmission dynamics between humans and cats to prevent future zoonotic threats and protect human and animal health.

The rapid spread of SARS-CoV-2 in densely populated urban areas like Lisbon, where humans and their pets live in proximity, provides a unique context for studying the virus’ transmission between human and animals. This research was conducted from November 2021 to March 2022 in Lisbon, focused on cats from 50 households with confirmed COVID-19 cases. Out of the 76 cats included in the study, 23 cats (30.3%) tested positive for IgG antibodies against SARS-CoV-2 receptor-binding domain (RBD) alpha, delta and omicron variants by ELISA. Subsequently, 16 of these 23 cats (69.6%) showed neutralizing antibodies against the same variants in a plate-based neutralization assay sVNT and against SARS-CoV-2 pseudotyped viral particles. Crossing the results from the ELISA and sVNT, 21% (16/76) of the animals were exposed to SARS-CoV-2 and developed neutralizing antibodies against the virus. ELISA, sVNT and SARS-CoV-2 pseudovirus neutralization assays were essential to confirming the specificity of cat antibodies against SARS-CoV-2 and providing a detailed understanding of the immune response in cats. These assays ensured that positive results indicated not only the presence of antibodies against the virus but also their ability to neutralize viral infection. Together, these complementary approaches enhance the robustness and reliability of our results. These results indicate high seroprevalence of SARS-CoV-2 in cats from COVID-19 positive households, suggesting a considerable level of exposure to the virus in these animals. It is important to note that the study period coincided with the circulation of the Delta variant (B.1.617.2) and the early emergence of the Omicron variant (BA.1) in Portugal. Our results showed that most cats, as indicated by ELISA, had antibodies against both the Delta and Omicron variants. However, a higher proportion of samples neutralized the Delta variant, while a more limited neutralizing response was observed for Omicron, with only four samples showing neutralization activity against this variant. These findings reflect the evolving landscape of circulating variants during the study period, suggesting that the observed neutralization capacity in cats may have been influenced by the prevalence of each variant at the time.

When compared to a previous study conducted by our group in Lisbon (December 2019 to October 2021), which focused on cats with unknown exposure to SARS-CoV-2 (38), the current study found a higher prevalence of antibodies (30.3% vs. 14.7%) and neutralizing antibodies (69.6% vs. 20.4%) in cats from COVID-19-positive households. Other studies, which also reported higher antibody prevalence in animals from households with confirmed COVID-19 cases, support our findings. For example, Kannekens-Jager et al. evaluated the seroprevalence of SARS-CoV-2 in households containing at least one individual with a confirmed COVID-19 diagnosis, as well as in cats and dogs visiting a veterinary clinic. The findings revealed that in households with COVID-19 cases, 18.8% of the animals tested positive for the virus (with 27 dogs and 31 cats being affected). In contrast, the prevalence was significantly lower among cats and dogs visiting the veterinary clinic, at just 4.6% (six dogs and nine cats tested positive) (16). According to another survey, 21 to 53% of cats and dogs in households with COVID-19 positive individuals showed a high prevalence of antibodies against SARS-CoV-2 (17). A study conducted in the US, which tested 1,000 dogs and cats exposed to human COVID-19 cases, found seropositivity rates of 33% in dogs and 27% in cats (41). Similar, a study conducted in COVID-19 positive households in Germany found seroprevalence rates of 42.5% in cats and 56.8% in dogs (42). These findings contribute to the growing body of literature suggesting that companion animals, particularly those living in COVID-19 positive households, are at a higher risk of SARS-CoV-2 exposure and seroconversion. Furthermore, another study that collected serum and swab samples from dogs and cats in homes with SARS-CoV-2 positive individuals, found seroprevalence rates of 37.5% in dogs and 28.6% in cats for anti-SARS-CoV-2 IgG. This study also reported low rates of positive SARS-CoV-2 PCR results in animals (43). This may reflect the short detection window due to rapid recovery or low viral loads in animals. This reinforces the idea that seropositivity, as a marker of prior exposure, can be a more reliable and accessible method for monitoring past infection compared to active viral detection.

Another aim of the present study was to elucidate the potential correlations and risk factors associated with SARS-CoV-2 seropositivity in cats, thereby providing a broader understanding of the dynamics of viral transmission in domestic settings. To achieve this, factors related to the animals’ lifestyle, owner-cat interactions and owners’ symptoms during infection period were evaluated. Our results revealed no statistically significant association between the risk factors studied and sVNT results. However, factors such as sex, owner-cat interactions (e.g., sharing food and changing litter), and more severe symptoms in owners (such as fever, loss of smell and taste, chills, and fatigue) could warrant further investigation in larger studies to better understand their potential role in SARS-CoV-2 transmission to cats.

Previous studies have identified several factors that may influence the risk of SARS-CoV-2 human-to-cat transmission. A higher number of infected individuals within the household, increased contact intensity, and behaviors such as co-sleeping with owners have been reported as significant risk factors for cat infection (11, 16, 42, 44). Additionally, factors such as shorter length of outdoor access and higher frequency of droppings removed from litter boxes were also significantly associated with higher infection rates (45). Unfortunately, limited studies exist on risk factors influencing SARS-CoV-2 transmission from humans to cats. This highlights the urgent need for further research to gain a better understanding of SARS-CoV-2 human-to-cat transmission dynamics and to develop effective strategies for preventing and controlling future zoonotic outbreaks.

One of the limitations of our study was the inability to perform SARS-CoV-2 pseudovirus neutralization assays for the delta and omicron variants due to limited sample availability. This limitation prevented us from fully validating the immune response to these variants and establishing a direct comparison between them. Furthermore, the absence of viral sequencing prevented a comparison of the viral variants circulating in both humans and cats. Although all seropositive cats in our study lived exclusively indoors, except for one with limited outdoor access, this does not provide definitive proof that infection occurred within the household. The absence of viral sequencing prevents us from determining whether the source of infection was the owner or another route such as contact with fomites or other animals. However, given the limited external exposure of these cats, human-to-cat transmission remains the most plausible explanation. Future studies including viral sequencing and a broader assessment of risk factors, will be essential for providing a comprehensive understanding of SARS-CoV-2 transmission dynamics.

## 5 Conclusion

The main objective of this study was to assess the seroprevalence of SARS-CoV-2 in cats from COVID-19-positive households. Our findings reveal that 30.3% of the cats in these households were positive for IgG antibodies against SARS-CoV-2 variants (alpha, delta and omicron). Significantly, 69.6% of these seropositive cats had neutralizing antibodies able to block the RBD-ACE2 interactions, giving additional support that these animals not only had been exposed to the virus but had mounted a protective immune response. Most cat serum samples neutralized SARS-CoV-2 alpha and delta variants, however the neutralizing activity against the omicron variant was lower. This indicates that although the immune response to SARS-CoV-2 was robust against the earlier variants, its effectiveness against newer variants may be compromised. These results highlight the ongoing evolution of the virus and its implications for both human and animal health. No statistical associations were found between specific risk factors and seropositivity. However, factors such as sex, owner-cat interactions (e.g., sharing food and changing litter), and more severe symptoms in owners (such as fever, loss of smell and taste, chills, and fatigue) could warrant further investigation in larger studies to better understand their potential role in SARS-CoV-2 transmission to cats. Future research needs to be directed toward identifying specific risk factors that facilitate human-to-cat transmission, including cat’s lifestyle and health conditions, owner-cat interactions and symptoms exhibited by owners. Furthermore, studies that include viral sequencing would help determine whether the source of infection is the owner or external exposure through contaminated surfaces or contact with other animals. This work underscores the relevance of ongoing studies focused on the interspecies transmission of SARS-CoV-2. This understanding is crucial in formulating effective management strategies to prevent the emergence of future zoonoses and mitigate risks to human and animal health.

## Data Availability

The original contributions presented in the study are included in the article/Supplementary material, further inquiries can be directed to the corresponding author.
