# Supplementary material for: Seroprevalence of SARS-CoV-2 in cats from COVID-19 positive households in the Lisbon area
Source: Front Vet Sci. 2025 Jun 23;12:1542397. doi: 10.3389/fvets.2025.1542397 (PMC12231644; doi:10.3389/fvets.2025.1542397)
Supplement: Supplementary file 1 [file Table_1.docx]

| **Risk Factors** | **Seropositive** | **Seronegative** |  |  |
| --- | --- | --- | --- | --- |
|  |  |  | **Bivariate Analysis** | |
|  |  |  | **p-value** | **OR**  **(CI 95%)** |
| **Sex:**  Male  Female | 5/76  11/76 | 31/76  29/76 | **0.**1656 | 0.097  [0.004 - 2.619] |
| **Age:**  Junior  Adult  Geriatric | 5/76  10/76  1/76 | 21/76  34/76  5/76 | 0.3752  0.3530 | 0.056  [9.674×10^-5^ - 32.602]  0.138  [2.126×10^-3^ - 8.995] |
| **Breed:**  Purebred  Mixed breed | 1/76  15/76 | 4/76  56/76 | 0.4134 | 0.104  [0.0005 - 23.453] |
| **Origin:**  Private  Other | 5/76  11/76 | 16/76  44/76 | 0.4590 | 3.673  [0.117 - 114.993] |
| **Retrovirus**  Positive  Negative  Unknown | -  13/76  3/76 | 1/76  42/76  17/76 | 0.7389  1.000 | 1.913  [0.042 - 86.750]  2.458×10^-10^  [0.000 - Inf] |
| **FIP**  Positive  Negative  Unknown | -  13/76  3/76 | -  43/76  17/76 | 0.7369 | 1.797  [0.059 - 54.847] |
| **Core Vaccination**  **Yes:**  **No:** | 10/76  6/76 | 33/76  27/76 | 0.741 | 0.578  [0.022 - 14.953] |
| **FeLV Vaccination**  **Yes:**  **No:** | 9/76  7/76 | 18/76  42/76 | 0.4394 | 2.935  [0.192 - 44.988] |
| **Lifestyle:**  Indoor  Outdoor | 16/72  - | 58/76  2/76 | 0.946 | 2.800×10^10^  [9.622×10^-294^ - Inf] |
| **Outdoor Access**  **Yes:**  **No:** | 1/76  15/76 | 13/76  47/16 | 0.3162 | 0.073  [0.0004 – 12.154] |
| **Sharing space with other animals**  **Yes:**  **No:** | 14/76  2/76 | 43/76  17/76 | 0.2736 | 11.961  [1.407×10^-1^ – 1016.415] |

| **Risk Factors** | **Seropositive** | **Seronegative** |  |  |
| --- | --- | --- | --- | --- |
|  |  |  | **Bivariate Analysis** | |
|  |  |  | **p-value** | **OR** |
| **Infected people in the household**  1  >1 | 3/76  13/76 | 27/76  33/76 | 0.2562 | 8.467  [2.120×10^-1^ - 338.199] |
| **Owner-Cat Interactions** | | | | |
| **Petting**  **Yes:**  **No:** | 15/76  1/76 | 53/76  7/76 | 0.631 | 4.122  [1.279×10^-2^ - 1328.815] |
| **Providing lap time**  **Yes:**  **No:** | 15/76  1/76 | 50/76  10/76 | 0.398 | 9.921  [4.856×10^-2^ - 2026.646] |
| **Giving licks**  **Yes:**  **No:** | 13/76  3/76 | 32/76  28/76 | 0.2031 | 12.509  [2.555×10^-1^ - 612.369] |
| **Playing**  **Yes:**  **No:** | 16/76  - | 51/76  9/76 | 0.983 | 7.722×10^10^  [0 - Inf] |
| **Sharing food**  **Yes:**  **No:** | 6/76  10/76 | 8/76  52/76 | 0.1775 | 32.436  [2.064×10^-1^ - 5097.068] |
| **Co-sleeping**  **Yes:**  **No:** | 16/76  - | 44/76  16/76 | 0.964 | 9.442×10^9^  [0 - Inf] |
| **Change Litter**  **Yes:**  **No:** | 15/76  1/76 | 44/76  16/76 | 0.1794 | 21.626  [2.432×10^-1^ - 1922.682] |
| **No interaction**  **Yes:**  **No:** | -  16/76 | 1/76  59/76 | 0.9877 | 1.929×10^-10^  [0 – Inf] |
| **Owners’ Symptoms** | | | | |
| **Fever**  **Yes:**  **No:** | 15/76  1/76 | 31/76  29/76 | 0.1060 | 1.528×10^2^  [3.433×10^-1^ - 68049.173] |
| **Cough**  **Yes:**  **No:** | 15/76  1/76 | 44/76  16/76 | 0.264 | 15.706  [1.249×10^-1^ 1974.153] |
| **Runny nose**  **Yes:**  **No:** | 11/76  5/76 | 31/76  29/76 | 0.457 | 3.706  [1.175×10^-1^ - 116.903] |
| **Loss of smell**  **Yes:**  **No:** | 11/76  5/76 | 18/76  42/76 | 0.1117 | 42.036  [4.200×10^-1^ - 4207.278] |
| **Loss of taste**  **Yes:**  **No:** | 11/76  5/76 | 20/76  40/76 | 0.1371 | 36.236  [3.188×10^-1^ - 4119.214] |
| **Chills**  **Yes:**  **No:** | 8/76  8/76 | 23/76  37/76 | 0.1020 | 3.220×10^6^  [5.102×10^-2^ - 2.032×10^14^] |
| **Vomiting**  **Yes:**  **No:** | 1/16  15/76 | 8/76  52/76 | 0.6473 | 0.265  [0.0008 - 78.449] |
| **Diarrhea**  **Yes:**  **No:** | 3/76  13/76 | 17/76  43/76 | 0.9044 | 0.786  [0.015 - 40.060] |
| **Shortness of breath**  **Yes:**  **No:** | 5/76  11/76 | 9/76  51/76 | 0.4537 | 4.699  [0.082 - 269.016] |
| **Fatigue**  **Yes:**  **No:** | 15/76  1/76 | 33/76  27/76 | 0.1090 | 69.053  [3.890×10^-1^ - 12258.186] |
| **Muscle pain**  **Yes:**  **No:** | 11/76  5/76 | 31/76  29/76 | 0.3649 | 5.806  [1.293×10^-1^ - 260.693] |
| **Sore throat**  **Yes:**  **No:** | 10/76  6/76 | 33/76  27/76 | 0.6303 | 2.321  [0.075 - 71.581] |
| **Headache**  **Yes:**  **No:** | 12/76  4/76 | 36/76  24/76 | 0.2658 | 12.117  [1.497×10^-1^ - 980.756] |
| **Chest pain**  **Yes:**  **No:**  **No response:** | 1/76  14/76  1/76 | 9/76  51/76  - | 0.2747 | 0.060  [0.0003 - 9.346] |
| **Abdominal**  **Pain**  **Yes:**  **No:** | 5/76  11/76 | 9/76  51/76 | 0.3373 | 9.979  [0.091 - 1096.384] |
| **Join pain**  **Yes:**  **No:** | 6/76  10/76 | 15/76  45/76 | 0.4132 | 5.414  [0.095 - 309.519] |
| **Dermatological changes**  **Yes:**  **No:** | -  16/76 | 3/76  57/76 | 1.000 | 5.329×10^-11^  [0.00 - Inf] |
| **Asymptomatic**  **Yes:**  **No:** | -  16/76 | 6/76  54/76 | 1.000 | 9.798×10^-11^  [0.00 - Inf] |
